# Durability of ChAdOx1 nCoV-19 (AZD1222) vaccine and hybrid humoral immunity against variants including omicron BA.1 and BA.4 6 months after vaccination (COV005): a post-hoc analysis of a randomised, phase 1b–2a trial

**DOI:** 10.1016/S1473-3099(22)00596-5

**Published:** 2023-03

**Authors:** Shabir A Madhi, Gaurav Kwatra, Simone I Richardson, Anthonet L Koen, Vicky Baillie, Clare L Cutland, Lee Fairlie, Sherman D Padayachee, Keertan Dheda, Shaun L Barnabas, Qasim Ebrahim Bhorat, Carmen Briner, Khatija Ahmed, Parvinder K Aley, Sutika Bhikha, A E Bhorat, Aliasgar Esmail, Elizea Horne, Haajira Kaldine, Christian K Mukendi, Vimbai Sharon Madzorera, Nelia P Manamela, Mduduzi Masilela, S Tandile Hermanus, Thopisang Motlou, Nonkululeko Mzindle, Suzette Oelofse, Faeezah Patel, Sarah Rhead, Lindie Rossouw, Carol Taoushanis, Samuel van Eck, Teresa Lambe, Sarah C Gilbert, Andrew J Pollard, Penny L Moore, Alane Izu

**Affiliations:** a South African Medical Research Council Vaccines and Infectious Diseases Analytics Research Unit, Faculty of Health Sciences, University of the Witwatersrand, Johannesburg, South Africa; b African Leadership in Vaccinology Expertise, Faculty of Health Sciences, University of the Witwatersrand, Johannesburg, South Africa; c South African Medical Research Council Antibody Immunity Research Unit, Faculty of Health Sciences, University of the Witwatersrand, Johannesburg, South Africa; d Wits Reproductive Health and HIV Institute, Faculty of Health Sciences, University of the Witwatersrand, Johannesburg, South Africa; e Perinatal HIV Research Unit, Faculty of Health Sciences, University of the Witwatersrand, Johannesburg, South Africa; f National Institute for Communicable Diseases of the National Health Laboratory Services, Johannesburg, South Africa; g Setshaba Research Centre, Tshwane, South Africa; h Centre for Lung Infection and Immunity, Division of Pulmonology, Department of Medicine and UCT Lung Institute, University of Cape Town, South Africa; i Family Centre for Research with Ubuntu, Department of Paediatrics, Stellenbosch University, Cape Town, South Africa; j Soweto Clinical Trials Centre, Soweto, South Africa; k Faculty of Health Sciences, Department of Medical Microbiology, University of Pretoria, Pretoria, South Africa; l Division of Infection and Immunity, University College London, London, UK; m Oxford Vaccine Group, Department of Paediatrics, University of Oxford, Oxford, UK; n Jenner Institute, Nuffield Department of Medicine, University of Oxford, Oxford, UK

## Abstract

**Background:**

COVID-19 vaccine rollout is lagging in Africa, where there has been a high rate of SARS-CoV-2 infection. We aimed to evaluate the effect of SARS-CoV-2 infection before vaccination with the ChAdOx-nCoV19 (AZD1222) vaccine on antibody responses through to 180 days.

**Methods:**

We did an unmasked post-hoc immunogenicity analysis after the first and second doses of AZD1222 in a randomised, placebo-controlled, phase 1b–2a study done in seven locations in South Africa. AZD1222 recipients who were HIV-uninfected, were stratified into baseline seropositive or seronegative groups using the serum anti-nucleocapsid (anti-N) immunoglobulin G (IgG) electroluminescence immunoassay to establish SARS-CoV-2 infection before the first dose of AZD1222. Binding IgG to spike (anti-S) and receptor binding domain (anti-RBD) were measured before the first dose (day 0), second dose (day 28), day 42, and day 180. Neutralising antibody (NAb) against SARS-CoV-2 variants D614G, beta, delta, gamma, and A.VOI.V2, and omicron BA1 and BA.4 variants, were measured by pseudovirus assay (day 28, day 42, and day 180). This trial is registered with ClinicalTrials.gov, NCT04444674, and the Pan African Clinicals Trials Registry, PACTR202006922165132.

**Findings:**

Of 185 individuals who were randomly assigned to AZD1222, we included 91 individuals who were baseline seropositive and 58 who were baseline seronegative, in the final analysis. In the seropositive group, there was little change of anti-S IgG (and anti-RBD IgG) or neutralising antibody (NAb) titres at day 42 compared with at day 28. Anti-S (and anti-RBD) IgG geometric mean concentrations (GMCs) were higher throughout in the seropositive compared with the seronegative group, including at day 180 (GMCs 517·8 [95% CI 411·3–651·9] *vs* 82·1 [55·2–122·3] BAU/mL). Also D614G NAb geometric mean titres (GMTs) were higher in the seropositive group than the seronegative group, as was the percentage with titres of at least 185 (80% putative risk reduction threshold [PRRT] against wild-type–alpha COVID-19), including at day 180 (92·0% [74·0–99·0] *vs* 18·2% [2·3–51·8). Similar findings were observed for beta, A.VOI.V2, and gamma. For delta, BA.1, and BA.4, NAb GMTs and the proportion with titres above the PRRT were substantially higher in the seropositive compared with seronegative group at day 28 and day 42, but no longer differed between the groups by day 180.

**Interpretation:**

A single dose of AZD1222 in the general African population, where COVID-19 vaccine coverage is low and SARS-CoV-2 seropositivity is 90%, could enhance the magnitude and quality of antibody responses to SARS-CoV-2.

**Funding:**

The Bill & Melinda Gates Foundation, the South African Medical Research Council, the UK Research and Innovation, the UK National Institute for Health Research, and the South African Medical Research Council.

**Translation:**

For the Zulu translation of the abstract see Supplementary Materials section.

## Introduction

The effectiveness of adaptive immunity against SARS-CoV-2 induced by vaccination or infection differs by variants of concern (VOC).^1^ The magnitude of increase in antispike protein (anti-S) binding IgG and neutralising antibody (NAb) induced by vaccines were directly correlated with vaccine efficacy against symptomatic coronavirus disease 19 (COVID-19) due to the wild-type or alpha VOC in phase 3 efficacy trials.^2^ Furthermore, the proposed thresholds of anti-S IgG and NAb as surrogate for risk reduction against wild-type or alpha variant symptomatic COVID-19 is similar for the non-replicating chimpanzee adenovirus vector SARS-CoV-2 vaccine (ChAdOx1–nCoV19 or AZD1222), mRNA-1273 and non-replicating adenovirus 26 vector (Ad26CoV2.S) COVID-19 vaccines.^3^, ^4^ Antibodies induced by infection or vaccines could also attenuate the clinical course of SARS-CoV-2 infection through Fc effector functions such as antibody dependent cellular cytotoxicity (ADCC)^5^, whereby there is active lysis of infected cells following recognition of the cell membrane surface being bound by specific antibodies.

**Research in context**

**Evidence before this study**

Antibodies induced by SARS-CoV-2 infection or vaccines are correlated with prevention of infection as well as the attenuation of clinical severity of COVID-19 infection through virus neutralisation and Fc effector functions. The recombinant replication-defective chimpanzee adenovirus SARS-CoV-2 glycoprotein vaccine (AZD1222) is being widely used across Africa, where vaccine coverage remains low but approximately 90% of the population has been infected by SARS-CoV-2. Previous findings show that a single AZD1222 dose in previously SARS-CoV-2 infected individuals elicits similar antibody responses compared with two doses in SARS-CoV-2 naive individuals. There is little data on the durability, breadth, and capacity to mediate Fc effector functions of antibodies that result from AZD1222 vaccination following SARS-CoV-2 infection (hybrid immunity), particularly in the era of dominance of COVID-19 due to infections by the omicron variant of concern (VOC).

We did a literature search on PubMed for reports from Nov 1, 2020 up to Aug 21, 2022 using the keywords (“COVID-19” OR “SARS-CoV-2”) AND (“vaccine*” OR “vaccination*”) AND “Hybrid Immunity” AND “Omicron”, with no restrictions on language. We identified 51 studies reporting hybrid immunity after COVID-19 vaccination. We identified 16 observational studies reporting on hybrid immunity, which included analysis against the omicron VOC. COVID-19 vaccination, which used AZD1222, BBV152 (inactivated COVID-19 vaccine), BNT162.b2, and mRNA-1273 vaccines following infection with SARS-CoV-2 resulted in significantly higher anti-spike protein (anti-S), anti-receptor binding domain (anti-RBD), antibody concentration and neutralisation activity titres against wild-type virus compared with vaccine-only induced immunity. Also, hybrid immunity was associated with higher amounts of neutralising activity against omicron, albeit lower relative to wild-type virus, compared with those with vaccine-only induced immunity following two doses of AZD1222 or BBV152 and 2–3 doses of mRNA. The cross-sectional observational studies, however, varied in the number of vaccine doses (2–3), time period between last dose of vaccine, and sampling timepoint (21 to 224 days) and neutralising activity testing was restricted to wild-type virus, omicron BA.1 or BA.2, and delta.

**Added value of this study**

We expanded the observations of previous studies to include analysis of hybrid compared with AZD1222-only induced immunity on the longitudinal kinetics of anti-S, anti-RBD, antibody dependent cellular cytotoxicity (ADCC) and neutralising antibody activity against wild-type (D614G), beta, delta, A.VOI.V2, gamma, BA.1, BA.4, and SARS-CoV from 28 days after the first dose of AZD1222 through to 180 days. Similar to earlier observations, our data show little increase of anti-S IgG, anti-RBD IgG, neutralising antibody, or ADCC following a second dose of AZD1222 within 4 weeks of the first dose in individuals infected with SARS-CoV-2 before the first dose, compared with immune responses after the second AZD1222 dose in those without SARS-CoV-2 infection before vaccination. Hybrid compared with vaccine-only induced immunity showed increased ability to neutralise VOCs, including the highly immune-evasive omicron BA.1 and BA.4 sub-lineages, which have dominated global infections in the first half of 2022, as well as SARS-CoV. Furthermore, neutralising antibodies titres remained higher at 142 days after the second dose (D180) in individuals with hybrid compared with vaccine-only induced immunity against many VOCs, but not for delta, BA.1, or BA.4.

**Implications of all the available evidence**

The low increase in antibody responses in individuals after a second dose of AZD1222 in those infected with SARS-CoV-2 before the first dose of vaccine, calls into question the utility of a second dose as soon as 4 weeks after the first dose in populations with a high previous rate of SARS-CoV-2 infection. Furthermore, hybrid immunity was associated with heightened neutralising activity against all VOCs including BA.1 and BA.4. Although there was waning of antibody neutralising activity against Delta, BA.1 and BA.4 by day 180 including in those with hybrid immunity, there is likely to be memory B cell responses against these variants in individuals with hybrid immunity. Our data indicate that even a single dose of AZD1222 at the general population level in Africa where there is high SARS-CoV-2 seropositivity and COVID-19 vaccine rollout is lagging, might be a valuable, cost-effective intervention to significantly enhance the magnitude and quality of antibody responses to SARS-CoV-2.

Infection with SARS-CoV-2 before COVID-19 vaccination (ie, hybrid immunity) is associated with robust anti-S IgG and NAb against wild-type virus after a single dose of AZD1222, AD26.CoVs.S, and mRNA (ie, BNT162.b2 and mRNA-1273) vaccines.^6^, ^7^, ^8^, ^9^ A second dose of AZD1222 or mRNA vaccines within 3–8 weeks after the initial dose in individuals previously infected by SARS-CoV-2, results in only modest further increase of anti-S IgG and NAb titres. In contrast, a second dose of AZD1222 and more markedly so for mRNA vaccines in individuals who were SARS-CoV-2 naive at time of vaccination, induces a multifold increase in anti-S IgG and NAb titres.^10^, ^11^ The anti-S IgG and NAb titres following a single dose of AZD1222 or mRNA in individuals vaccinated after SARS-CoV-2 infection are greater or equal with the antibody responses following two doses of the homologous vaccine in SARS-CoV-2 naive individuals.^12^, ^13^, ^14^

COVID-19 vaccine and infection induced protection against severe COVID-19 is potentially mediated by T-cell induced immunity, which is less affected by mutations in the VOCs that have evolved to date.^15^ Nevertheless, heightened magnitude of NAb induced by hybrid immunity compared with immunity only induced by COVID-19 vaccines or SARS-CoV-2 infection, could better protect against non-severe COVID-19 even in infections caused by VOCs with NAb evasive mutations.^16^

There is little knowledge on the effects of hybrid immunity on NAb against a range of variants, including against omicron BA.1 and BA.4. Furthermore, there is a paucity of data on the effect of hybrid immunity on durability of NAb against wild-type virus and subsequent variants or against SARS-CoV, by 180 days after vaccination. COVID-19 vaccination using AZD1222, BBV152 (inactivated COVID-19 vaccine), BNT162.b2, and mRNA-1273 messenger RNA (mRNA) vaccines following infection with SARS-CoV-2 was associated with higher concentrations of NAb against omicron, albeit lower against wild type, compared with vaccine-only induced immunity following two doses of AZD1222 or BBV152 and 2–3 doses of mRNA.^17^, ^18^, ^19^, ^20^ The cross-sectional observational studies, however, varied in the number of vaccine doses (2–3), time period between last dose of vaccine, and sampling timepoint (21–224 days) and NAb testing was restricted to wild-type virus, omicron BA.1 or BA.2, and delta.^17^, ^18^, ^19^, ^20^

The main focus of this analysis was to evaluate the effect of SARS-CoV-2 infection before the first dose of AZD1222 on the post-vaccine antibody kinetics through to day 180. We report on the antibody kinetics of anti-S IgG, anti-receptor binding domain (anti-RBD) IgG, NAb against wild type, multiple VOCs (including omicron BA.1, BA.4) and SARS-CoV, and Fc effector function to wild-type and delta VOC.

## Methods

### Study design and participants

We analysed samples collected in a randomised, placebo-controlled, phase 1b–2a trial, which evaluated the safety, immunogenicity, and efficacy of AZD1222. Details of the multicentre study (COV005) done in South Africa have been described, including interim analysis of vaccine efficacy and immunogenicity through to 14 days following the two doses of AZD1222 in people living with HIV and HIV-negative individuals.^21^, ^22^ Participants were enrolled into the study between June 24, 2020 and Nov 9, 2020. The study protocol, detail of the inclusion and exclusion criteria, and randomisation is available online. The study was approved by the South African Health and Pharmaceutical Products Regulatory Authority and the Human Ethics Research Committees of the various sites. Signed informed consent was obtained from all study participants, which included consent for further testing of samples not included in the original protocol.

### Randomisation and masking

Healthy adults aged 18–65 years who were eligible for study participation were randomly assigned (1:1) via a computer-generated system with full allocation concealment, to receive two intramuscular injections of either AZD1222 or saline placebo (0·9% sodium chloride; placebo group), given 28 days apart.^21^, ^22^ Sample size for the study was determined on the basis of the primary efficacy objective and a total sample size of 1907 would provide sufficient power to detect vaccine efficacy of at least 60% (with the lower limit of a 95% CI for vaccine efficacy greater than 0%, assuming an attack rate of 3·5% in placebo participants). Enrolment of the initial cohort of 70 HIV-uninfected individuals included in the phase 1b study occurred between June 24, 2020 and July 29, 2020; and the rest (n=1956) were enrolled through to Nov 9, 2020.

There was a higher than anticipated percentage of HIV-uninfected participants (322 [19%] 1683) without a documented history of COVID-19 but who tested positive for anti-nucleocapsid (anti-N) IgG at time of randomisation (hereafter referred to as the seropositive group). Consequently, we did a post-hoc analysis of durability of antibody responses comparing AZD1222 recipients with and without SARS-CoV-2 infection before receipt of the first dose. Sampling timepoints for measuring antibody responses included the day of the first study injection (day 0), at the time of the second dose of study injection (day 28), 14 days after the second dose (day 42) and 6 months post study entry (day 180).

**Figure 1:**
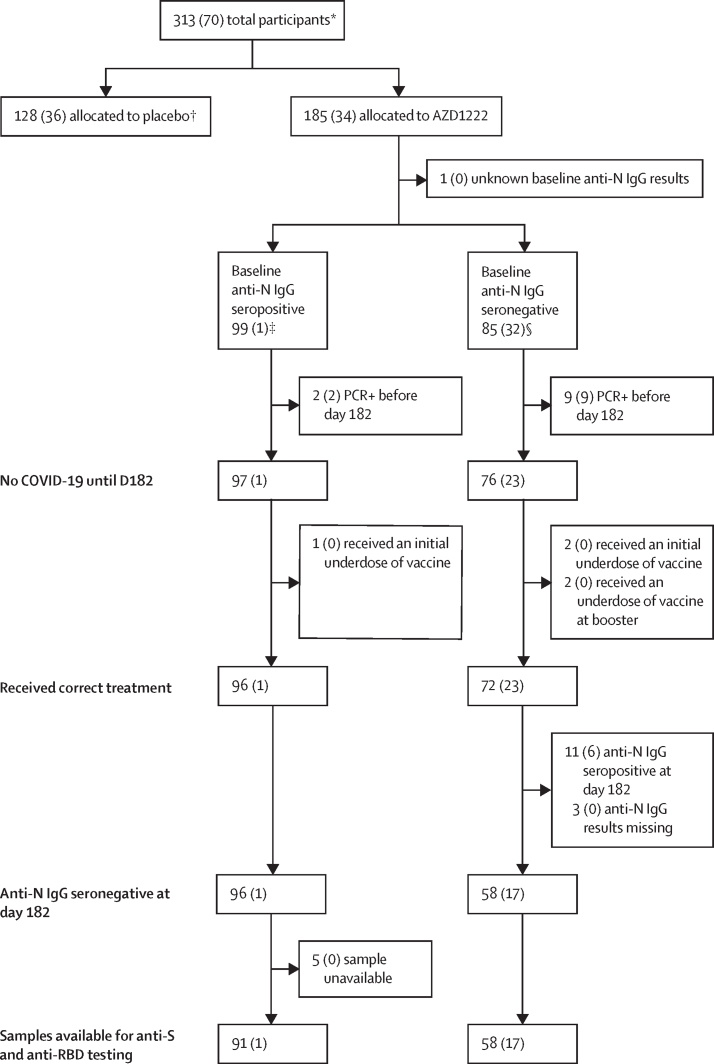
Trial profile Number of participants enrolled in the phase 1b study is shown in parenthesis. *Neutralisation assays were done on all phase 1b participants and an additional random sample of phase 2 vaccinated baseline anti-N IgG seropositive participants. In total, 41 vaccinated participants (17 from phase 1b) and nine placebo participants (all phase 1b) had neutralisation assays done. †The antibody kinetics of the placebo group is not reported further in this article. ‡There were 25 vaccinated participants who were anti-N IgG seropositive at baseline who had neutralisation results (only one from phase 1b). All 25 participants with baseline anti-N IgG seropositive results were included in the analysis of the neutralisation data. §There were 16 phase 1b vaccinated participants who were anti-N IgG seronegative at baseline who had neutralisation results; of these, two had PCR^+^ results before day 182 and three had an anti-N IgG seropositive response at day 182. A total of 11 vaccinated participants with baseline anti-N IgG seronegative results were included in the analysis of the neutralisation data. Anti-N=anti-nucleocapsid. Anti-S=anti-spike protein. Anti-RBD=anti-receptor binding domain.

The immunogenicity (anti-RBD and anti-S IgG and NAb against Asp614Gly wild-type [D614G] and beta variant only) of AZD1222 in people living with HIV through to day 42, including stratified by prevaccination SARS-CoV-2 infection status, and immunogenicity through to day 42 in a smaller subset of HIV-uninfected individuals who were SARS-CoV-2 naive (26 placebo and 27 AZD1222 recipients) enrolled in phase 1b has been previously published.^22^ 17 of the AZD1222 recipients included in the phase 1b study are also included in the current analysis. For this report, we expanded testing of immune responses in the HIV-uninfected cohort to an additional 243 participants enrolled into phase 2 of the study, who were randomly selected by use of a random number generating function after stratification by baseline anti-N IgG serostatus. We aimed to include approximately 75 AZD1222 recipients who were baseline anti-N IgG seropositive and 75 who were seronegative at enrolment. Participants with any intercurrent SARS-CoV-2 infection between enrolment and day 180, whether diagnosed by nucleic acid amplification test or anti-N IgG seroconversion by day 180, were excluded from the final analysis (figure 1). This was a descriptive, hypothesis-generating study and did not include any sample size calculation.

### Procedures

Binding antibodies were measured by singleplex bead-based immunoassays on the Luminex platform to quantify serum IgG binding to full length spike and RBD of wild type; and reported in binding antibody units per millilitre (BAU/mL) as described (appendix 2 p 1).^22^ The anti-RBD and anti-S (full length spike) assay was calibrated against a research reagent for anti-SARS-CoV-2 antibody (code 20/130 supplied by National Institute for Biological Standards and Control, Herts, UK). Anti-N IgG was measured using the Roche Elecsys anti-SARS-CoV-2 serology test is an electroluminescence immunoassay-based modality that allows for the qualitative detection of IgG reactive to the SARS-CoV-2 nucleocapsid protein in human sera, as previously described.^21^

Neutralising antibody responses were evaluated in a convenience subset of samples, which screened positive for anti-S IgG and for which sufficient residual sample was available for pseudovirus neutralising antibody assay (PSVN) testing. Pseudovirus neutralisation assays were as described.^23^ Briefly, the SARS-CoV-2 Wuhan-1 spike, cloned into pCDNA3.1 was mutated using the QuikChange Lightning Site-Directed Mutagenesis kit (Agilent Technologies, Santa Clara, CA, USA) and NEBuilder HiFi DNA Assembly Master Mix (Ipswich, MA, USA) to include D614G (original) or lineage defining mutations for beta, delta, omicron BA.1, omicron BA.4, A.VOI.V2, and gamma with the mutations further detailed in the appendix 2 (pp 1–2). We also assayed SARS-CoV, which differs from SARS-CoV-2 by approximately 30% in its sequence identity, to assess neutralisation cross-reactivity. Pseudotyped lentiviruses were prepared by co-transfecting the HEK293T cell line with the spike plasmids in conjunction with a firefly luciferase encoding lentivirus backbone (HIV-1 pNL4.luc) plasmid. Culture supernatants were clarified of cells by 0·45-μmol/L filter and stored at −70°C. For the neutralisation assay, plasma samples were heat-inactivated and clarified by centrifugation, then incubated with the SARS-CoV-2 pseudotyped virus for 1 h at 37°C with 5% CO_2_. Subsequently, 1 × 10^4^ HEK293T cells engineered to over-express ACE-2 (293T–ACE2.MF; kindly provided by M. Farzan (Scripps Research La Jolla, CA, USA) were added and incubated at 37°C with 5% CO_2_ for 72 h after which the luminescence of the luciferase protein was measured. Titres were calculated as the reciprocal plasma dilution (ID_50_) causing 50% reduction of relative light units. Titres were calculated as the reciprocal plasma dilution (ID_50_) causing 50% reduction of infection.

### Outcomes

We did a post-hoc analysis of durability of antibody responses comparing AZD1222 recipients with and without SARS-CoV-2 infection before receipt of the first dose. Another post-hoc study objective was evaluation of ADCC through to day 180 in a convenience subset of 30 samples, which screened positive for anti-S IgG and for which sufficient residual sample was available. Because of the highly intensive nature of the ADCC assay, testing was limited to targeting D614G and delta on the basis of previous observation that Fc effector function is conserved across variants.^24^ Details of the ADCC assay have been previously described and are summarised in the appendix 2 (p 2).

### Statistical analysis

Geometric mean concentrations (GMCs) and 95% CIs were calculated for anti-S IgG, anti-RBD IgG, and ADCC IgG. Pseudovirus NAb geometric mean titres (GMTs) and 95% CIs were evaluated at day 28, day 42, and day 180. Titres below the lower limit of detection (LoD) were given the value of half the LoD. We calculated 95% CIs for GMCs or GMTs by back transforming the 95% CI for log antibody concentrations or titres.

We analysed the proportion of individuals with an anti-S, anti-RBD IgG concentration and PSVN NAb titre above the threshold predictive of 80% risk reduction of symptomatic COVID19 (referred to as putative risk reduction threshold [PRRT]) due to wild type and mainly alpha VOC using the approach described by Feng and colleagues for the AZD1222 vaccine.^25^ The proposed thresholds predictive of 80% risk reduction against wild-type or alpha VOC (referred to as wild-type–alpha) symptomatic COVID-19 were: anti-S IgG of 264 BAU/mL, anti-RBD IgG of 506 BAU/mL and PSVN NAb titre of 185. Variant-specific NAb thresholds associated with risk reduction of symptomatic COVID-19 for variants emerging since alpha VOC have not been established. Consequently, we used the wild-type–alpha PSVN NAb PRRT threshold for the other VOCs. We used the Wilson method to calculate 95% CI for proportions.^26^

The increase factor between day 0 and day 28 of NAb titres was evaluated by presenting the geometric mean of the ratio of NAb titres at day 28 and day 0. The geometric mean of the ratio of Nab titres of D6414G and each variant was calculated at all timepoints. The 95% CI for geometric mean ratios were calculated by back transforming the 95% CI for the log of the ratio.

The percentage reduction in geometric means of NAb titres between day 42 and day 180 are calculated as subtracting the geometric mean of the ratio of NAb titres at day 180 and day 42 from one. 95% CI for percentage reduction in geometric means were calculated by subtracting the 95% CI for the geometric mean ratio from one.

Comparisons of demographic and clinical features between baseline anti-N IgG seropositive and seronegative individuals were done by means of Wilcoxon rank sum test for quantitative variables and χ^2^ or Fisher's exact test for categorical variables. For binding and neutralisation results, GMCs were compared using *t* test and binary variables were compared using Fisher's exact test. p values less than 0·05 were considered significant.

This is a descriptive study and no considerations were taken into account for multiple testing or adjustment for differences in demographics between the two groups.

The COV005 study is registered with ClinicalTrials.gov, NCT04444674, and the Pan African Clinical Trials Registry, ACTR202006922165132.

### Role of the funding source

The funders of the study had no role in study design, data collection, data analysis, data interpretation, or writing of the report. The partners of the study reviewed the data from the trial and the final manuscript before submission, but the authors had final responsibility to submit for publication.

## Results

For this study, we expanded testing of immune responses in the HIV-uninfected cohort enrolled in phase 2 and included 313 participants (128 placebo and 185 AZD1222 recipients). We do not report further on the kinetics of antibodies in the placebo group in this manuscript. After exclusions, 149 AZD1222 participants remained eligible in the final analysis, 99 of whom were anti-N seropositive before the first dose of AZD1222 and 58 of whom were seronegative and remained free of SARS-CoV-2 infection through to day 180 (figure 1). Overall, median age was younger in the baseline seropositive (28; IQR 24–37 years) than the seronegative group (35; IQR 26–44 years) and a higher percentage of the baseline seropositive group were Black (76 [84%] of 91 *vs* 45 [78%] of 58). Other demographic characteristics were similar between the seropositive and seronegative groups, with 81 (54·%) of 149 being male. The median time following the second dose of AZD1222 at which immunogenicity was evaluated was 14 (IQR 14–14) days; and persistence of antibody was measured through to a median of 148 (IQR 143–154) days following the second dose of AZD1222 (table).

**Table:** Baseline characteristics

		**Anti-N IgG seropositive group (n=91)**	**Anti-N IgG seronegative group (n=58)**	p value*
Female		43 (47)	25 (43%)	0·74
Male		48 (53%)	33 (57%)	..
Age, years		28 (24–37)	35 (26–44)	0·015
Body-mass index		..	..	0·91
	Underweight	4 (4%)	4 (7%)	..
	Normal	42 (46%)	25 (43%)	..
	Overweight	25 (27%)	17 (29%)	..
	Obese	20 (22%)	12 (21%)	..
Smoker		41 (45%)	27 (47%)	0·99
Alcohol		50 (55%)	27 (47%)	0·41
Health worker		4 (4%)	4 (7%)	0·71
Race		..	..	0·011
	Black	76 (84%)	45 (78%)	..
	Mixed race	13 (14%)	4 (7%)	..
	White	1 (1%)	6 (10%)	..
	Other	1 (1%)	3 (5%)	..
Hypertension		2 (2%)	1 (2%)	>0·99
Respiratory system		2 (2%)	2 (3%)	0·64
Diabetes		1 (1%)	0	>0·99
HbA_1c_ level†		..	..	>0·99
	Low	0	1	..
	Normal	1	16	..
	High	0	0	..
Time between doses, days		28 (28–33)	28 (28–29)	0·27
Time 2 weeks after second dose, days		14 (14–14)	14 (14–14)	0·79
Time 6 months after second dose, days		149 (144–154)	146 (142–154)	0·89

Data are n (%) and median (IQR).

* p value comparing characteristics between baseline antiN IgG seropositive and baseline anti-N IgG seronegative individuals.

† Measured in safety cohort only.

As expected, anti-S IgG GMCs (binding antibody units [BAU]/mL) at day 0 was higher in the seropositive (183·2; 95% CI 137·7–243·8) compared with the seronegative group (1·1; 0·8–1·5; p<0·0001), although only 37 (43%) of 87 of the seropositive group had antibody concentrations above PRRT for symptomatic wild-type–alpha COVID-19. At day 28, anti-S GMCs remained higher in the seropositive (2040·5; 1591·5–2616·3) compared with seronegative group (136·4; 102·2–182·0 BAU/mL), as was the percentage of individuals with anti-S IgG above the PRRT against wild-type–alpha (82 [92%] of 89 *vs* 14 [25%] of 55; p<0·0001).

Between day 28 and day 42, there was no significant increase in anti-S IgG GMC in the seropositive group, but a 4·1 times increase in the seronegative group. Nevertheless, the day 42 anti-S IgG GMCs remained higher in the seropositive group (2194·9 [95% CI 1574·2–3060·2] *vs* 538·4 [421·9–687·0] BAU/mL). Furthermore, even the day 28 anti-S IgG GMC in the seropositive group (2040·5 [1591·5–2616·3] BAU/mL) was higher than the day 42 peak GMCs in the seronegative group (538·4 [421·9–687·0] BAU/mL). The percentage of individuals with anti-S IgG above the PRRT against wild-type–alpha COVID-19 at day 42 remained higher in the seropositive (85 [97%] of 88) compared with the seronegative group (40 [74%] of 54; p<0·0001).

**Figure 2:**
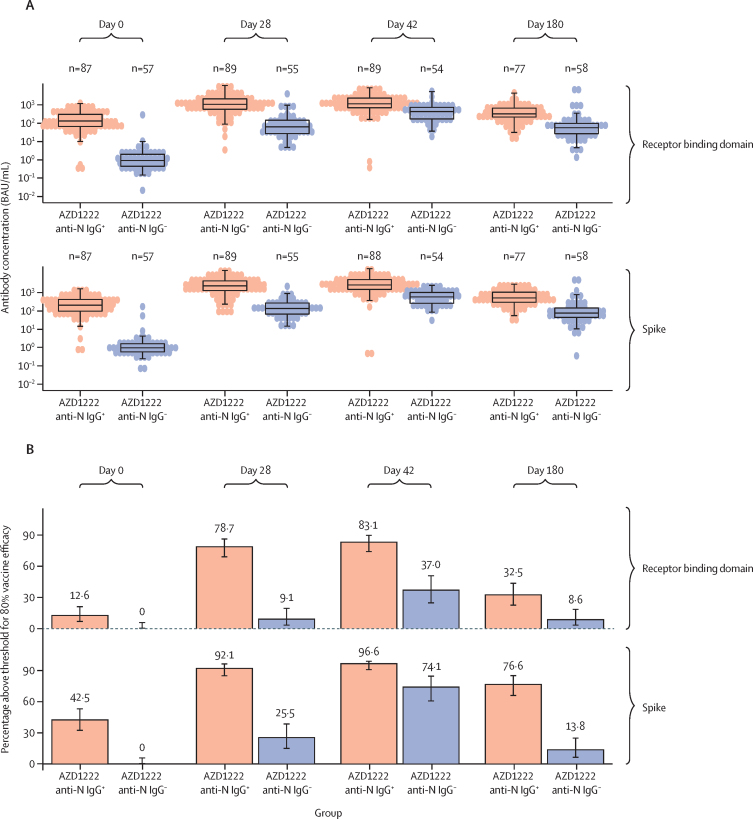
Antibody kinetics comparing AZD1222 vaccinated individuals who were anti-nucleocapsid IgG seropositive or seronegative at time of first dose of vaccine (A). Percentage of participants with anti-receptor binding domain IgG and anti-Spike IgG concentrations above the threshold for 80% risk reduction against wild-type or alpha variant of concern,* comparing AZD1222 vaccinated individuals who were anti-nucleocapsid IgG seropositive (anti-N IgG^+^) or seronegative (anti-N IgG^−^) at time of first dose of vaccine (B) BAU=binding antibody units. Anti-N=anti-nucleocapsid. *The putative binding IgG threshold associated with 80% risk reduction of wild-type or alpha variant of concern symptomatic COVID-19 was as determined by Feng and colleagues;^25^ and was 264 binding arbitrary units per millilitre (BAU/mL) for anti-spike IgG and 506 for antireceptor binding domain IgG.

Between day 42 and day 180, there was less of a decline in anti-S IgG GMC in the seropositive (73·5%; 95% CI 63·7–80·7%) compared with the seronegative group (84·7%; 77·9–89·4%); and anti-S IgG GMC remained higher in the seropositive group (517·8 [411·3–651·9] *vs* 82·1 [77·9–89·4] BAU/mL). Furthermore, the percentage of individuals with anti-S IgG above PRRT against wild-type–alpha COVID-19 remained higher in the seropositive (59 [77% of 77]) compared with the seronegative group (8 [14%] of 58; p<0·0001) at day 180. The day 180 anti-S IgG GMCs in the seropositive group (517·8; 95% CI 411·3–651·9) was similar to the day 42 peak GMCs in the seronegative group (538·4; 421·9–687·0 BAU/mL); as was the percentage of people with IgG concentrations above the PRRT against wild-type–alpha COVID-19 (59 [77%] of 77] *vs* 40 [74%] of 54], respectively; figure 2A–B; appendix 2 p 4).

The dynamics of anti-RBD IgG and differences between the seropositive and seronegative groups were similar to that observed for anti-S IgG. Generally, the percentage of individuals with IgG greater than the PRRT against wild-type–alpha symptomatic COVID-19 was lower for anti-RBD IgG than anti-S IgG (figure 2A–B; appendix 2 p 4.

The ADCC IgG GMT was higher in the seropositive group compared with the seronegative group at day 0 against D614G (499·9 [95% CI 362·5–683·9] *vs* 46·1 [18·9–112·7] relative light units (RLUs) and delta variant (382·4 [295·0–495·9] *vs* 37·9 [17·5–82·2] RLU). There was a greater increase factor in ADCC IgG GMT in the seronegative compared with seropositive group by day 28 against D614G (9·6 *vs* 1·6 times increase) and delta variant (9·0 *vs* 1·7 times). Nevertheless, the ADCC IgG GMT remained higher in the seropositive compared with the seronegative group for D614G (807·8 [635·4–1026·8] vs 440·2 [293·6–66·1]) and delta (636·6; 516·5–784·7 *vs* 297·2; 183·4–481·5) at day 28.

Between day 28 and day 42, there was no further increase in ADCC IgG GMT in the seropositive group, compared with 2·3 and 2·7 times increase against D614G and the delta variant, respectively, in the seronegative group. Consequently, there was no difference in ADCC IgG GMT against D614G or delta between the seropositive compared with the seronegative group at day 42. Also, the ADCC IgG GMT at D180 did not differ by baseline sero status for either D614G or delta (appendix 2 p 5). The geometric mean of the ADCC IgG D614G to delta ratio was similar between the seropositive and seronegative vaccine recipients at each timepoint and ranged between 1·1 and 1·5 (appendix 2 p 5).

**Figure 3:**
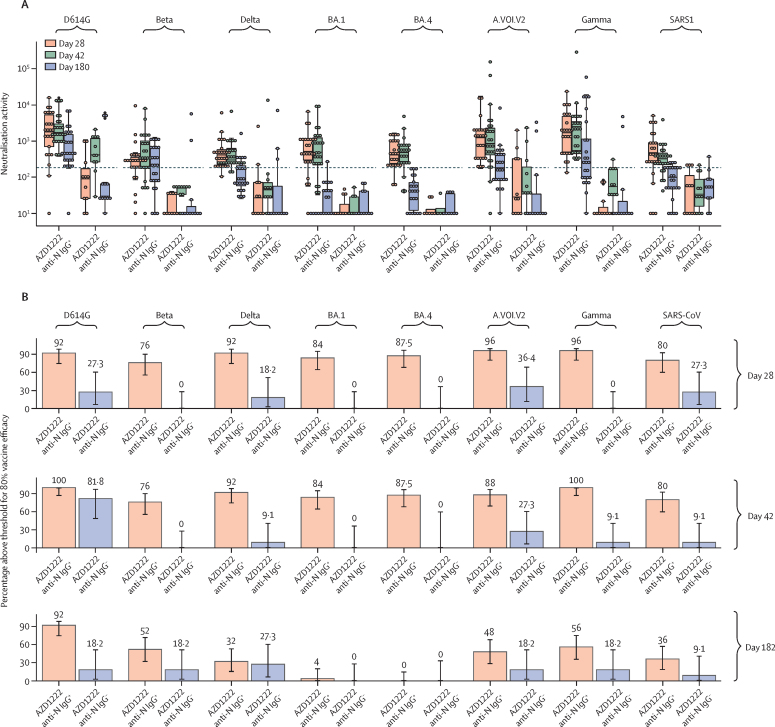
Pseudovirus neutralisation antibody activity to multiple variants 1 month after first AZD1222 dose (day 28), 14 days (day 42) and 142 days (day 180) the second dose of AZD1222, stratified by baseline anti-nucleocapsid IgG seropositive (anti-N IgG^+^) or seronegative (anti-N IgG^−^) at time of first vaccine dose (A). Examining the percentage of participants with the reciprocal plasma dilution ID_50_ concentrations above the putative threshold for 80% risk reduction* of symptomatic COVID-19 in AZD1222 vaccinated individuals who were anti-nucleocapsid IgG seropositive (anti-N IgG^+^) or seronegative (anti-N IgG^−^) at time of first dose of vaccine (B) Anti-N=anti-nucleocapsid. *The putative pseudovirus neutralisation assay (PSVN) antibody titre of 185 associated with 80% risk reduction of wild-type or alpha variant of concern symptomatic COVID-19 was as determined by Feng and colleagues.^25^ We presumed that the same threshold would apply for PSVN for the other variants, and SARS-CoV-1.

PSVN GMTs against D614G were higher at days 28, 42, and 180 in the seropositive group compared with the seronegative group. Also, a higher percentage of the baseline seropositive group (23 [92%] of 25) than the seronegative group (three [27%] of 11; p<0·0002) had NAb above the PRRT against wild-type–alpha symptomatic COVID-19 at day 28. The GMTs against D614G increased 5·6 times between day 28 (81; 95% CI 28–237) and day 42 (451; 197–1035) in the seronegative group, whereas there was only a 1·3 times increase (1496; 768–2916 to 1933; 1283–2912) in the seropositive group. Nevertheless, GMT was 4·3 times higher in the seropositive group compared with the seronegative group at day 42; and the percentage with above the PRRT was higher in the seropositive group, albeit not significant (25 [100%] of 25] *vs* nine [82%] of 11], p=0·087). The NAb titres against D614G decreased by 69·5% (95% CI 43·7–83·5) and 82·6% (25·1–96·0) in the seropositive and seronegative groups, respectively, between day 42 and day 180; and GMT remained higher in the seropositive group (590 [95% CI 337–1032] *vs* 78 [19–329]). By day 180, the seropositive group were again more likely (23 [92%] of 25) to have NAb above the PRRT than the seronegative group (2 [18%] of 11; p<0·0001; figure 3A–B; appendix 2 p 6).

The NAb GMT against D614G was 1·5 to 12·8 times higher than against the beta VOC at the different time points. At day 28, 19 (76%) of 25 of the seropositive compared with none of the seronegative group had NAb above the PRRT against beta symptomatic COVID-19. There was only a modest increase factor in NAb against beta between day 28 and day 42 in the seropositive (1·5) and seronegative (2·2) groups. The day 180 NAb GMT against beta in the seropositive group (178; 95% CI 96–329) was 5·1 times higher than the peak at day 42 in the seronegative group (35; 23–55). The percentage of individuals with NAb titres greater than the PRRT against beta symptomatic COVID-19 was consistently higher in the seropositive group than the seronegative group at days 28 and 42 (19 [76%] of 25] *vs* 0% at both points; p<0·0001), with a similar trend observed at day 180 (13 [52%] of 25] *vs* two [18%] of 11]; p=0·077; figure 3A–B; appendix 2 p 6).

The NAb kinetics against A.VOI.V2, gamma and SARS-CoV between the seropositive and seronegative group, as well as percentage at each timepoint above the PRRT threshold, were similar to that observed for beta VOC (figure 3A–B; appendix 2 pp 9–11).

The NAb GMT against D614G was 2·1 to 7·4 times higher than against delta at the different timepoints. Against delta variant, there was no significant increase in NAb GMTs after the second dose of AZD1222 (between days 28 and 42) in the baseline seropositive or seronegative group, however, GMT remained higher in the seropositive group (416; 95% CI 294–588 *vs* 61; 16–226; p=0·0088) at day 42. The day 180 NAb GMT titre against delta in the seropositive group (112; 75–168) was similar to the day 42 peak NAb GMT in the seronegative group. The percentage of individuals with NAb above the PRRT against delta symptomatic COVID-19 was consistently higher in the seropositive group than the seronegative group at day 28 (23 [92%] of 25 *vs* two [18%] of 11; p<0·0001), and day 42 (23 [92%] of 25 *vs* one [9%] of 11; p<0·0001), whereas it was similar at day 180 (eight [32%] of 25 *vs* three [27%] of 11; p=0·99; figure 3A–B; appendix 2 p 6).

The NAb GMT against D614G was 3·0 to 27·4 times higher than against omicron BA.1. At day 28, the NAb GMT against BA.1 was higher in the seropositive group (499; 95% CI 282–885) than the seronegative group (14; 9–22). There was no increase in NAb against BA.1 in either the seropositive group or seronegative group following the second dose of AZD1222, and NAb GMTs remained higher in the seropositive group at day 42 (535; 290–988 *vs* 16; 9–29). The percentage of individuals with NAb above the PRRT against BA.1 symptomatic COVID-19 was 21 (84%) of 25 at day 28 and day 42 in the seropositive group, and 0% in the seronegative group (p<0·0001). There was a 96·0% (92·4–97·9) reduction in BA.1 NAb GMT between day 42 and day 180 in the seropositive group, with only one (4·0%) of 25 of them still having titres above the PRRT at day 180 (figure 3A–B; appendix 2 p 8).

Kinetics of NAbs against omicron BA.4 mirrored omicron BA.1. NAb GMTs against BA.4 were higher in the seropositive group compared with the seronegative group at all timepoints. For the seropositive group, NAb GMTs decreased over time, whereas NAb GMTs ranged from 13 (95% CI 9–19) to 16 (9–27) between day 28 and day 180 in the seronegative group. The percentage of individuals with NAb above the PRRT against BA.4 symptomatic COVID-19 was 21 (88%) of 24 at day 28 and day 42 in the seropositive group, and 0% in the seronegative group. At day 180, no participants had a titre above the putative threshold and there was a 90·4% (80·9–95·2) decrease in the NAb GMT from day 42 in the seropositive group.

## Discussion

The findings of our study show that SARS-CoV-2 infections, before vaccination primed the immune system for a more robust humoral immune response to the AZD1222 primary series compared with individuals who were SARS-CoV-2 naive when vaccinated. Similar observations were reported by other studies on AZD1222 vaccine as an effective booster after initial priming with SARS-CoV-2 infection.^27^

Furthermore, a second dose of AZD1222 within 4 weeks of the first dose in individuals who were baseline seropositive, did not result in a further increase in anti-S IgG, anti-RBD IgG, Nab, or ADCC. In contrast there were substantial increases in antibody concentrations following the second dose of AZD1222 in individuals who were seronegative at baseline. Similar observations were reported by others after the primary series for AZD1222 for anti-S and NAb to wild type virus,^28^ as well as for mRNA and Ad26.CoV2.v2.^6^, ^29^ However, we expand the observations to include the implications of hybrid-induced immunity compared with AZD1222-only induced immunity on kinetics of anti-S, anti-RBD, and ADCC through to day 180. Furthermore, we analysed the effect of hybrid immunity induced neutralising antibody activity to several SARS-CoV-2 variants, including omicron BA.1 and BA.4, which dominate global infections in 2022.

The baseline anti-N IgG seropositive compared with seronegative AZD1222 recipients maintained higher concentrations of anti-S and anti-RBD IgG through to day 180. Also, a greater percentage of vaccine recipients with hybrid immunity had NAb above the PRRT against symptomatic COVID-19 due to D614G, beta, A.VOI.V2, and gamma through to day 180. Although a higher percentage of the seropositive group compared with the seronegative group also had NAb titres above the PRRT against symptomatic COVID-19 due to delta (92·0% *vs* 9·1%), BA.1 (84% *vs* 0%) and BA.4 (88% *vs* 0%) at day 42, this was no longer evident by day 180. Nevertheless, there is likely to be memory B cell responses in individuals with hybrid immunity against delta, BA.1, and BA.4. The antibody-evasiveness of omicron BA.1 and BA.4 has been well described for convalescent sera and COVID-19 vaccines.^30^, ^31^, ^32^, ^33^

The higher potential vaccine effectiveness even against variants showing relative resistance to NAb activity imputed from our analysis, is corroborated from real world vaccine effectiveness in which hybrid immunity with mRNA vaccines had higher vaccine effectiveness against BA.1 and BA.2 infection (52–57%) compared with only two doses of mRNA vaccine induced immunity.^34^ The higher hybrid immunity induced vaccine effectiveness could be attributable to the greater magnitude of binding and NAb induced after only a single dose of vaccine compared with even the peak antibody responses at day 42 in the seronegative group. Also, the repertoire of immune responses following vaccination after past infection compared with in a SARS-CoV-2 naive individual might vary in terms of antibody targets and degree of affinity maturation and through a broader repertoire of CD4^+^ and CD8^+^ immune responses.^35^ Antibody mediated Fc effector function evaluated here, albeit in a small subset, showed that infection alone (at day 0), as well as vaccine-only induced immunity elicited cross-reactive ADCC against D614G and delta; with the ADCC IgG showing less waning through to day 180 compared with binding IgG and NAb concentrations. This is consistent with previous reports of antibodies retaining the ability to mediate Fc effector function against VOCs.^24^

We previously reported from the same study the lack of vaccine efficacy against mild–moderate symptomatic COVID-19 due to the beta variant (vaccine efficacy 10%; 95% CI −79 to 55%) in individuals who were anti-N IgG seronegative at enrolment. The lack of AZD1222 vaccine effectiveness against beta is consistent with our observation that none of the baseline seronegative group had NAb above the PRRT against beta variant symptomatic COVID-19 at day 42. In contrast, the percentage with NAb above the PRRT in the seropositive group was 71·7% at day 28 and remained high through to day 180 (51·9%). This illustrates the need for exercising caution in extrapolating from studies done early on in the pandemic when a lower percentage of the populations were infected by SARS-CoV-2 compared with the present when the population is broadly SARS-CoV-2 experienced.^36^

Notably, most individuals with hybrid immunity (92% at day 28 and day 42) had NAb against delta titres above the PRRT compared with only 9% of those with vaccine-only induced immunity. The final analysis of study recorded vaccine efficacy of 77·1% (95% CI 30·4–94·4) against the delta variant, with the cases having accrued 9–10 months after the second dose of AZD1222, independent of baseline anti-N IgG serostatus; and vaccine efficacy against wild-type virus was 91% (35–100).^37^ We have not completed anti-N IgG at all the timepoints as a proxy for SARS-CoV-2 infections over and above the cases documented by nucleic acid amplification test positivity. Nevertheless, a serosurvey before the delta variant wave in several South African provinces showed SARS-CoV-2 seropositivity rates of 32–63% by January, 2021.^38^ This could have contributed to a high degree of protection against delta, which dominated in South Africa between May, 2021, to early November, 2021, despite the low NAb activity induced by vaccine alone.

We had unmasked the study and offered dosing of the placebo group and a third dose to the vaccinated group before the onset of the omicron dominant wave. Consequently, we cannot extrapolate whether the high percentage of individuals with hybrid immunity who had NAb above the PRRT against BA.1 at day 42 (84%) translated into vaccine effectiveness against mild–moderate COVID-19 due to omicron BA.1. Notably, 70% of COVID-19 unvaccinated adults in Gauteng Province (South Africa), where most sites involved in this study were based, were seropositive before the omicron dominant wave in South Africa.^21^

Limitations of our study include that we focused only on vaccine and infection induced binding and NAb responses, which are important contributors to protecting against SARS-CoV-2 infection and mild-moderate COVID.^2^, ^5^ We did not evaluate CD4^+^ and CD8^+^ T lymphocyte responses, which probably attenuate the clinical severity of COVID-19. Notably, CD4^+^ and CD8^+^ T-cell responses induced by vaccines and past infection are preserved, even against the highly mutated omicron BA.1 variant harbouring more than 30 S-protein mutations.^39^ Another limitation was use of the PSVN threshold associated with 80% risk reduction of wild-type–alpha symptomatic COVID-19, as a PRRT for other analysed variants, for which individual thresholds have not yet been established. Also, we did not measure anti-N IgG at day 28 and day 42, but rather used SARS-CoV-2 nucleic acid amplification test confirmed cases identified from active surveillance and sampling at routine scheduled visits to exclude infections between day 0 and day 180. Consequently, we might have inadvertently missed some episodes of SARS-CoV-2 infections. Nevertheless, as the focus of the study was to establish the effect of antecedent SARS-CoV-2 infection on immune responses to the AZD1222 vaccine, our study findings would be generalisable to a real world situation where undocumented exposures to SARS-CoV-2 occur. Our study was also limited by the number of samples that were available for NAb or ADCC testing, and there were some differences in median age and race between the groups, which we do not adjust for in the analysis. Also, the baseline seropositive group was infected with the wild-type virus and it's unclear whether primary infection with other variants would yield similar observations as we have shown.

Overall, these data indicate that vaccine effectiveness against symptomatic COVID-19 is likely to differ at an individual and population level depending on previous SARS-CoV-2 infection. Also, the low boost in antibody responses following a second dose of a vaccine in previously infected individuals suggests a low value at the population level (excluding immunocompromised individuals) of a second dose of vaccine 4 weeks later in settings where there has been a high rate of past SARS-CoV-2 infections. It is, however, possible that a longer interval between the first two doses could confer benefit in individuals with past SARS-CoV-2 infection. This is pertinent in many low-income African countries where COVID-19 vaccine rollout is still lagging, but 79% of the population had been infected with SARS-CoV-2 by mid-November, 2021, before the circulation of the highly transmissible omicron variant.^40^

## Data sharing

Deidentified individual-level participant data and data dictionary will be available for sharing after approval of a proposal by the University of the Witwatersrand Human Ethics Research Committee and following a signed data access agreement. Requests for data sharing can be directed to the corresponding author.

## Declaration of interests

Oxford University has entered into a partnership with AstraZeneca for further development of ChAdOx1 nCoV-19 (AZD1222). SCG is cofounder of Vaccitech, a collaborator in the early development of this vaccine candidate, and is named as an inventor on a patent covering use of ChAdOx1-vectored vaccines (PCT–GB2012–000467) and a patent application covering this SARS-CoV-2 vaccine (GB2003670.3). TL is named as an inventor on a patent application covering ChAdOx1 nCoV-19 and was a consultant to Vaccitech. All other authors declare no competing interests.

## Supplementary Materials

Zulu translation of the abstract
