# Supplementary material for: Durability of ChAdOx1 nCoV-19 (AZD1222) vaccine and hybrid humoral immunity against variants including omicron BA.1 and BA.4 6 months after vaccination (COV005): a post-hoc analysis of a randomised, phase 1b–2a trial
Source: Lancet Infect Dis. 2023 Mar;23(3):295–306. doi: 10.1016/S1473-3099(22)00596-5 (PMC9584570; doi:10.1016/S1473-3099(22)00596-5)
Supplement: Zulu translation of the abstract [file mmc1.pdf]

THE LANCET
Infectious Diseases

Supplementary appendix 1

This translation in isiZulu was submitted by the authors and we reproduce it as supplied. It has not been peer reviewed. *The Lancet*'s editorial processes have only been applied to the original in English, which should serve as reference for this manuscript.

Lokhu kuhumusha ngesiZulu kwethulwe ngababhali futhi siyikhiqiza kabusha njengoba kuhlinzekiwe. Akuzange kubuyekezwe ontanga. Izinqubo zokuhlela zeLancet zisetshenziswe kuphela koqobo lwesiNgisi, okufanele lusebenze njengesethenjwa salo mbhalo.

Supplement to: Madhi SA, Kwatra G, Richardson SI, et al. Durability of ChAdOx1 nCoV-19 (AZD1222) vaccine and hybrid humoral immunity against variants including omicron BA.1 and BA.4 6 months after vaccination (COV005): a post-hoc analysis of a randomised, phase 1b–2a trial. *Lancet Infect Dis* 2022; published online Oct 20. https://doi.org/10.1016/S1473-3099(22)00596-5.

Sesotho Translation:

Lipatlisiso tsa tšoarello ea tširelletso ea ente ea Chadox-nCoV19 (ADZ1222) khahlanong le mefuta ea-COVID e kang Omicron BA.1 and BA.4.6: a post-hoc analysis of a randomised, phase 1b-2a trial.

[Durability of the combined Chadox vaccine immunity against other variants, including omicron, 6 months after vaccination. This is an analysis post the phase 1b-2a trial.]

Khutshufatso [Summary]

Bo kamorao [Background]:

Qalo ea ente ea COVID-19 ea khoehla Africa, moo ho na le sekhahla se phahameng sa tšoaetso se ngata sa SARS-COV-19. Re ne re ikemeselitse ho hlahloba phello ea tšoaetso ho karabelo ea li-antibody (masole a mmele)pele ho fumana ente ea Chadox-nCoV19(AZD1222). Re tle be re hlahoba karabelo ea teng matsatseng a 180.

Mokhoa [Methods]:
Ka mora ente ea pele le ea bobeli ea ADZ1222, ka hara "randomised placebo-controlled phase 1b-2a" phuputsong e entsoeng libakeng tse supileng Africa-Boroa. Re entse tlhahlobo e pepesitsoeng ea litlamorao tsa tširelletso ea ente.
Baamoheli ba ente AZD1222 ba ne ba se na Phamokate ba hlophuoe ka sehlopha sa seronegative (sehlopha sa bao ba seke ba ba le tsoaetso ea COVID) le seropositive(Sehlopha sa bao ba ileng ba ba le tšoaetso ea COVID) ho sebelisoa teko ea serum 'anti-nucleocapsid (anti-N) immunoglobulin G (IgG) electroluminescence immunoassay" ho theha tšoaetso ea SARS-CoV19 pele ho ente ea pele ea AZD1222. Re lekantsitse 'Binding IgG to spike (anti-S)' le 'receptor binding domain (anti-RBD) li likantsoe pele ho ente ea pele ea AZD1222 (day 0: letsatsi la pele), ente ea bobedi (day 28), le ka mora matsatsi a 42 (day 42) le ka mora matsatsi a 180 (day 180).' Mefuta ea SARS-CoV19 e leng: D614G, beta, delta, gamma le A.VOI.V2, and omicron BA1 le BA.4 li likantose ka 'pseudovirus assay' ka (day 28, day 42, le day 180) khahlano le 'Neutralising antibody (NAb)'. Patlisiso ena e ngodisitsoe ho le ClinicalTrials.gov, NCT04444674, le the Pan African Clinicals Trials Registry, PACTR202006922165132.

Tse Fumanoeng [Findings]
Ha ra batho ba 185 ba ne ba hlophetsoe AZD1222, re kenyelelitse batho ba 91 shelopeng sa 'Seropositive' le ba 58 sehlopheng sa 'Seronegative' hlahlobeng ea ho qetela. Ho sehlopha se Seropositive: ho no honale phethoho e nyane ea Anti-S IgG (le Anti-RBD) le neutralising sntibody (Nab) ka (day 42) ha ho bapisoa le ka day 28.
Anti-S (le anti-RBD) IgG 'geometric mean concentrations (GMC)' di ne di le hodimo hohle sehlopheng se Seropositive ha ho bapisoa le shehlopha se SeroNegative ho kenyeletsoa le ka day 180. Hape le D614G NAb geometric mean titres (GMTs) di ne di le hodimo ho sehlopha se Seropositive ho feta sehlopha se SeroNegative. Ho joalo le ka peresente ea titre ea bonyane ba 185 (80% putative risk reduction threshold [PRRT] against wild-type–alpha COVID-19) ho kenyeletsoa le ka day 180 (92·0% [74·0–99·0] *vs* 18·2% [2·3–51·8). Lipatlisiso tse tšoanang di tholahetsoe ka for beta, A.VOI.V2, le gamma . ho Delta BA.1 and BA.4, NAb, tekanyo ea li-titres tse ne di feta li- putative risk reduction threshold [PRRT] dine dile hodimo sehlopheng se Seropositive ho feta sehlopheng se Seronegative ka day 28 le day 42. Empe ho ne ho sena phaphang mahareng a li-hlopha ka day 180.

Tlhaloso [Interpretation]

Ente elengoe ea AZD1222 ho kakaraetso ea batho ba Africa moo kgaso ea ente ea COVID-19 e leng tlase le SARS-CoV-2 seropositivity e leng peresente tse 90, e ka matlafatsa boholo le boleng ba karabao ea masole a mmele ho SARS-CoV-2.

Tsa dichelte [Funding]
The Bill & Melinda Gates Foundation, South African Medical Research Council, the UK Research and Innovation, the UK National Institute for Health Research, le South African Medical Research Council.

© 2022 The Bangoli. Published by Elsevier Ltd. This is an Open Access article under the CC BY 4.0 license.

Sš
