# Supplementary material for: Durability of ChAdOx1 nCoV-19 (AZD1222) vaccine and hybrid humoral immunity against variants including omicron BA.1 and BA.4 6 months after vaccination (COV005): a post-hoc analysis of a randomised, phase 1b–2a trial
Source: Lancet Infect Dis. 2023 Mar;23(3):295–306. doi: 10.1016/S1473-3099(22)00596-5 (PMC9584570; doi:10.1016/S1473-3099(22)00596-5)
Supplement: Supplementary appendix 2 [file mmc2.pdf]

THE LANCET
Infectious Diseases

Supplementary appendix 2

This appendix formed part of the original submission and has been peer reviewed. We post it as supplied by the authors.

Supplement to: Madhi SA, Kwatra G, Richardson SI, et al. Durability of ChAdOx1 nCoV-19 (AZD1222) vaccine and hybrid humoral immunity against variants including omicron BA.1 and BA.4 6 months after vaccination (COV005): a post-hoc analysis of a randomised, phase 1b–2a trial. *Lancet Infect Dis* 2022; published online Oct 20. https://doi.org/10.1016/S1473-3099(22)00596-5.

Supplementary material

Durability of ChAdOx1 nCoV-19 (AZD1222) vaccine and hybrid humoral immunity against variants including omicron BA.1 and BA.4 6 months after vaccination (COV005): a post-hoc analysis of a randomised, phase 1b–2a trial

Methods of the anti-spike (ant-s) and anti-receptor domain (anti-RBD) immunoglobulin G assays

Binding antibodies were measured by singleplex bead-based immunoassays on the Luminex platform to quantify serum IgG binding to full length spike and RBD of the WT virus; and reported in binding antibody units per millilitre (BAU/ml) as described[1]. The recombinant RBD and spike proteins were expressed as described previously [2] and was coupled to the magnetic microsphere beads (Bio-Rad, USA) using a two-step carbodiimide reaction [3]. An in-house references serum was developed by pooling convalescent serum from adult COVID-19 positive patients and was calibrated against a research reagent for anti-SARS-CoV-2 antibody (code 20/130 supplied by National Institute for Biological Standards and Control, Herts, UK). The binding antibody units (BAU) values assigned to in-house reference serum were 1242 BAU/mL and 2819 BAU/mL for RBD and full-length Spike IgG, respectively. The assay was also evaluated against a COVID-19 convalescent plasma panel (NIBSC code 20/118) intended for the development and evaluation of serological assays for the detection of antibodies against SARS-CoV-2. Samples were analysed in true duplicates and each plate included two in-house control sera. Bead fluorescence was read with the Bio-Plex 200 instrument (Bio-Rad) using Bio-Plex manager 5.0 software (Bio-Rad).

Methods of the pseudovirus neutralization assay and antibody dependent cellular cytoxic assays.

(Pseudotyped lentiviruses were prepared as previously described. Briefly, the SARS-CoV-2 Wuhan-1 spike gene sequence, cloned into pcDNA3.1, was mutated to include D614G (ancestral) or lineage defining mutations for Beta (L18F, D80A, D215G, Δ242-244, K417N, E484K, N501Y, D614G and A701V), Delta (T19R, Δ156-157, R158G, L452R, T478K,

D614G, P681R, D950N), Gamma (L18F, T20N, P26S, D138Y, R190S, K417T, E484K, N501Y, D614G, H655Y, T1027I, V1176F), A.VOI.V2 (D80Y, Δ144, I210N, Δ211, D215G, R246M, Δ247-249, W258L, R346K, T478R, E484K, H655Y, P681H, Q957H) and Omicron BA.1 (A67V, Δ69-70, T95I, G142D, Δ143-145, Δ211, L212I, EPE214, G339D, S371L, S373P, S375F, K417N, N440K, G446S, S477N, T478K, E484A, Q493K, G496S, Q498R, N501Y, Y505H, T547K, D614G, H655Y, N679K, P681H, N764K, D796Y, N856K, Q954H, N969K, L981F).

Antibody-dependent cellular cytotoxicity (ADCC) assay

The ability of plasma antibodies to cross-link and signal through FcγRIIIa (CD16) and spike expressing cells or SARS-CoV-2 protein was measured as a proxy for ADCC. For spike assays, HEK293T cells were transfected with 5μg of SARS-CoV-2 spike plasmids using PEI-MAX 40,000 (Polysciences) and incubated for 2 days at 37°C. Expression of spike was confirmed by differential binding of CR3022 and P2B-2F6 and their detection by anti-IgG APC staining measured by flow cytometry. Subsequently, $1x10^5$ spike transfected cells per well were incubated with heat inactivated plasma (1:100 final dilution) or monoclonal antibodies (final concentration of 100 μg/ml) in RPMI 1640 media supplemented with 10% FBS 1% Pen/Strep (Gibco, Gaithersburg, MD) for 1 hour at 37°C. Jurkat-Lucia™ NFAT-CD16 cells (Invivogen) ($2x10^5$ cells/well and $1x10^5$ cells/well for spike and other protein respectively) were added and incubated for 24 hours at 37°C, 5% CO_2. Twenty μl of supernatant was then transferred to a white 96-well plate with 50 μl of reconstituted QUANTI-Luc secreted luciferase and read immediately on a Victor 3 luminometer with 1s integration time. Relative light units (RLU) of a no antibody control was subtracted as background. Palivizumab was used as a negative control, while CR3022 was used as a positive control, and P2B-2F6 to differentiate the Beta from the D614G variant. 084-7D was used as a positive control for Omicron BA.1 and Beta. To induce the transgene 1x cell stimulation cocktail (Thermofisher Scientific, Oslo, Norway) and 2 μg/ml ionomycin in R10 was added as a positive control to confirm sufficient expression of the Fc receptor. RLUs for spikes were normalised to each other and between

runs using CR3022. All samples were run head to head in the same experiment as were all variants tested.

Suppl Table S1: Geometric mean concentrations at vaccination (day 0), second dose (day 28), two-weeks post 2nd dose (day 42) and six months post-vaccination (day 180) for anti- receptor binding domain IgG and anti-Spike IgG in AZD1222 vaccinated participants who were anti-Nucleoclapsid IgG sero-positive or sero-negative at baseline.

		anti-Receptor Binding Domain IgG			anti-Spike protein IgG		
Day	Measurement	AZD1222<br>anti-N IgG seropositive	AZD1222<br>anti-N IgG seronegative	p-value	AZD1222<br>anti-N IgG seropositive	AZD1222<br>anti-N IgG seronegative	p-value
D0	IgG GMCs[1]	116.6 (85.1,159.8) 87	1.0 (0.7,1.4) 57	<0.0001	183.2 (137.7,243.8) 87	1.1 (0.8,1.5) 57	<0.0001
	% > PRRT for 80% VE[2]	12.6 (7.2-21.2); 11/87	0.0 (0.0-6.3); 0/57	0.0034	42.5 (32.7-53.0); 37/87	0.0 (0.0-6.3); 0/57	<0.0001
D28	IgG GMCs[1]	957.7 (727.2,1261.2) 89	65.3 (44.7,95.6) 55	<0.0001	2040.5 (1591.5,2616.3) 89	136.4 (102.2,182.0) 55	<0.0001
	% > PRRT for 80% VE[2]	78.7 (69.0-85.9); 70/89	9.1 (3.9-19.6); 5/55	<0.0001	92.1 (84.6-96.1); 82/89	25.5 (15.8-38.3); 14/55	<0.0001
	Fold increase[3]	8.04 (6.09,10.63) 86	61.77 (39.52,96.55) 54	<0.0001	10.97 (8.35,14.40) 86	112.87 (77.28,164.85) 54	<0.0001
	≥4 fold increase[4]	74.4 (64.3-82.5); 64/86	94.4 (84.9-98.1); 51/54	0.0027	79.1 (69.3-86.3); 68/86	98.1 (90.2-99.7); 53/54	0.00079
D42	IgG GMCs[1]	1066.1 (782.5,1452.5) 89	379.0 (284.1,505.6) 54	<0.0001	2194.9 (1574.3,3060.2) 88	538.4 (421.9,687.0) 54	<0.0001
	% > PRRT for 80% VE[2]	83.1 (74.0-89.5); 74/89	37.0 (25.4-50.4); 20/54	<0.0001	96.6 (90.5-98.8); 85/88	74.1 (61.1-83.9); 40/54	<0.0001
	Fold increase[3]	1.06 (0.78,1.44) 88	6.54 (4.49,9.52) 51	<0.0001	1.04 (0.79,1.36) 87	4.10 (3.15,5.34) 51	<0.0001
	≥4 fold increase[4]	12.5 (7.1-21.0); 11/88	56.9 (43.3-69.5); 29/51	<0.0001	11.5 (6.4-19.9); 10/87	41.2 (28.8-54.8); 21/51	0.0001
D180	IgG GMCs[1]	306.4 (239.2,392.5) 77	58.0 (38.4,87.7) 58	<0.0001	517.8 (411.3,651.9) 77	82.1 (55.2,122.3) 58	<0.0001
	% >PRRT for 80% VE[2]	32.5 (23.1-43.5); 25/77	8.6 (3.7-18.6); 5/58	0.00084	76.6 (66.0-84.7); 59/77	13.8 (7.2-24.9); 8/58	<0.0001
	% decrease from Day 42[5]	70.2 (57.3,79.1) 76	84.9 (78.1,89.5) 54	0.0093	73.5 (63.7,80.7) 76	84.7 (77.9, 89.4) 54	0.025

[1]Geometric Mean Concentrations (GMCs) of IgG measurements with 95% confidence intervals shown within parenthesis followed by the sample size

[2]Percentage of participants with anti- receptor binding domain IgG and anti-Spike IgG concentrations above the putative risk reduction threshold (PRRT) associated with 80% vaccine efficacy (VE) followed by the sample size

[3]Geometric means of fold-increases are also reported at booster (day 28) and two-weeks post-2nd dose (day 42). 95% confidence intervals are shown within parenthesis. followed by the sample size

[4]Percentage of participants with more than a four-fold increase at 2nd dose (day 28) and two-weeks post 2nd dose (day 42). 95% confidence intervals are shown within parenthesis followed by the fraction of participants with more than a four-fold increase. .

[5]The percent reduction in the geometric means between two-weeks post 2nd dose (day 42) and six months post-vaccination (day 182). 95% confidence intervals are shown within parenthesis followed by the sample size.

Supplemental Table S2: Geometric mean concentrations at 1st vaccine dose (day 0), second vaccine dose (day 28), two-weeks post 2nd dose (day 42) and six months post-vaccination (day 180) of Antibody dependent cellular cytotoxicity relative light unit (FcγRIIIa signalling) in vaccinated baseline anti-N sero-positive and sero-negative participants.

Day	Measurement	D614G variant			Delta variant		
		AZD1222 anti-N IgG seropositive	AZD1222 anti-N IgG seronegative	p-value	AZD1222 anti-N IgG seropositive	AZD1222 anti-N IgG seronegative	p-value
D0	IgG GMT[1]	499.9 (362.5,689.3) 23	46.1 (18.9,112.7) 7	0.00028	382.4 (295.0,495.9) 23	37.9 (17.5,82.2) 6	0.00022
	D6414G/Delta[2]	Not applicable			1.3 (1.0,1.7) 23	1.1 (0.4,2.8) 6	0.69
D28	IgG[1]	807.8 (635.4,1026.8) 23	440.2 (293.6,660.1) 8	0.011	636.6 (516.5,784.7) 23	297.2 (183.4,481.5) 8	0.0068
	Fold increase[3]	1.6 (1.1,2.4) 23	9.6 (3.2,28.7) 7	0.0062	1.7 (1.4,2.0) 23	9.0 (4.6,17.5) 6	0.00069
	D6414G/Delta[2]	Not applicable			1.3 (1.1,1.5) 23	1.5 (1.0,2.3) 8	0.44
D42	IgG GMT[1]	959.6 (717.5,1283.4) 23	991.7 (626.3,1570.3) 8	0.89	685.1 (552.7,849.1) 23	792.4 (589.8,1064.6) 8	0.38
	Fold increase[3]	1.2 (0.95,1.5) 23	2.3 (1.2,4.3) 8	0.055	1.1 (0.9,1.3) 23	2.7 (1.3,5.4) 8	0.02
	D6414G/Delta[2]	Not applicable			1.4 (1.2,1.7) 23	1.3 (0.9,1.7) 8	0.46
D180	IgG GMT[1]	351.5 (192.2,642.6) 23	316.6 (161.9,618.9) 8	0.80	356.2 (195.9,647.6) 21	235.8 (96.5,575.8) 8	0.40
	% decrease from Day 42[4]	63.4 (25.47,82.00) 23	68.1 (27.14,86.01) 8	0.78	47.9 (3.64,71.85) 21	70.3 (27.97,87.71) 8	0.26
	D6414G/Delta[2]	Not applicable			1.2 (0.7,2.1) 21	1.3 (0.6,3.2) 8	0.81

[1]Geometric mean titres (GMT) of IgG measurements with 95% confidence intervals shown within parenthesis followed by the sample size

[2]The geometric mean of the ratio of D6414G and Delta (or equivalently the ratio of the geometric means of D6414G and Delta)are reported at all time points. 95% confidence intervals are shown within parenthesis followed by the sample size

[3]Geometric means of fold-increases are also reported at booster (day 28) and two-weeks post-booster (day 42). 95% confidence intervals are shown within parenthesis followed by the sample size.

[4]The percent reduction in the geometric means between two-weeks post-booster (day 42) and six months post-vaccination (day 182). 95% confidence intervals are shown within parenthesis followed by the sample size.

Supplementary Table S3: Pseudovirus neutralization antibody activity to multiple variants at 14 days (D42) and 142 days (D180) following second dose of AZD1222, stratified by baseline anti-Nucleocapsid IgG sero-positive or sero-negative at time of first dose of vaccine.

Variant	Day	Measurement	Anti-nucleocapsid IgG sero-positive at enrolment	Anti-nucleocapsid IgG sero-negative at enrolment	p-value
D614G	28	ID50 GMT[1] (95% CI); N	1496 (768,2916) n=25	81 (28,237) n=11	<0.0001
		Percentage > LoD[2] (95% CI) n/N	96.0 (79.6-99.9); 24/25	81.8 (48.2-97.7); 9/11	0.22
		% > PRRT[3] (95% CI) n/N	92.0 (74.0-99.0); 23/25	27.3 (6.0-61.0); 3/11	0.00020
	42	ID50 GMT[1] (95% CI); N	1933 (1283,2912) n=25	451 (197,1035) n=11	0.0033
		Percentage > LoD[2] (95% CI) n/N	100.0 (86.3-100.0); 25/25	100.0 (71.5-100.0); 11/11	>0.99
		% > PRRT[3] (95% CI) n/N	100.0 (86.3-100.0); 25/25	81.8 (48.2-97.7); 9/11	0.087
		Fold increase[4] (95% CI) N	1.3 (0.7,2.4) n=25	5.6 (2.6,12.0) n=11	0.0036
	180	ID50 GMT[1] (95% CI); N	590 (337,1032) n=25	78 (19,329) n=11	0.012
		Percentage > LoD[2] (95% CI) n/N	96.0 (79.6-99.9); 24/25	90.9 (58.7-99.8); 10/11	0.52
		% > PRRT[3] (95% CI) n/N	92.0 (74.0-99.0); 23/25	18.2 (2.3-51.8); 2/11	<0.0001
		% decrease from Day 42[5] (95% CI); N	69.5 (43.7,83.5) n=25	82.6 (25.1,96.0) n=11	0.45
Beta	28	ID50 GMT[1] (95% CI); N	248 (143,430) n=25	16 (10,26) n=11	<0.0001
		Percentage > LoD[2] (95% CI) n/N	96.0 (79.6-99.9); 24/25	36.4 (10.9-69.2); 4/11	0.00028
		% > PRRT[3] (95% CI) n/N	76.0 (54.9-90.6); 19/25	0.0 (0.0-28.5); 0/11	<0.0001
		D6414G/Beta[6] (95% CI); N	6.0 (3.3,11.1) n=25	5.0 (1.6,15.4) n=11	0.76
	42	ID50 GMT[1] (95% CI); N	370 (199,686) n=25	35 (23,55) n=11	<0.0001

		Percentage > LoD[2] (95% CI) n/N	96.0 (79.6-99.9); 24/25	81.8 (48.2-97.7); 9/11	0.22
		% > PRRT[3] (95% CI) n/N	76.0 (54.9-90.6); 19/25	0.0 (0.0-28.5); 0/11	<0.0001
		Fold increase[4] (95% CI) N	1.5 (0.9,2.4) n=25	2.2 (1.1,4.3) n=11	0.33
		D6414G/Beta[6] (95% CI); N	5.2 (2.8,9.8) n=25	12.8 (6.9,23.8) n=11	0.038
	180	ID50 GMT[1] (95% CI); N	178 (96,329) n=25	29 (7,132) n=11	0.028
		Percentage > LoD[2] (95% CI) n/N	88.0 (68.8-97.5); 22/25	27.3 (6.0-61.0); 3/11	0.00066
		% > PRRT[3] (95% CI) n/N	52.0 (31.3-72.2); 13/25	18.2 (2.3-51.8); 2/11	0.077
		% decrease from Day 42[5] (95% CI); N	51.8 (20.8,70.7) n=25	16.6 (-249.0,80.1) n=11	0.44
		D6414G/Beta[6] (95% CI); N	3.3 (1.7,6.3) n=25	2.7 (1.8,3.9) n=11	0.55
Delta	28	ID50 GMT[1] (95% CI); N	451 (316,643) n=25	38 (12,125) n=11	0.00080
		Percentage > LoD[2] (95% CI) n/N	100.0 (86.3-100.0); 25/25	54.5 (23.4-83.3); 6/11	0.0012
		% > PRRT[3] (95% CI) n/N	92.0 (74.0-99.0); 23/25	18.2 (2.3-51.8); 2/11	<0.0001
		D6414G/Delta[6] (95% CI); N	3.3 (1.6,6.9) n=25	2.1 (1.0,4.6) n=11	0.37
	42	ID50 GMT[1] (95% CI); N	416 (294,588) n=25	61 (16,226) n=11	0.0088
		Percentage > LoD[2] (95% CI) n/N	100.0 (86.3-100.0); 25/25	81.8 (48.2-97.7); 9/11	0.087
		% > PRRT[3] (95% CI) n/N	92.0 (74.0-99.0); 23/25	9.1 (0.2-41.3); 1/11	<0.0001
		Fold increase[4] (95% CI) N	0.9 (0.7,1.1) n=25	1.6 (0.6,3.9) n=11	0.22
		D6414G/Delta[6] (95% CI); N	4.6 (3.1,7.1) n=25	7.4 (2.6,20.9) n=11	0.37
	180	ID50 GMT[1] (95% CI); N	112 (75,168) n=25	36 (8,172) n=11	0.15
		Percentage > LoD[2] (95% CI) n/N	100.0 (86.3-100.0); 25/25	27.3 (6.0-61.0); 3/11	<0.0001

		% > PRRT[3] (95% CI) n/N	32.0 (14.9-53.5); 8/25	27.3 (6.0-61.0); 3/11	>0.99
		% decrease from Day 42[5] (95% CI); N	73.0 (54.8,83.9) n=25	40.4 (-191.4,87.8) n=11	0.31
		D6414G/Delta[6] (95% CI); N	5.3 (2.9,9.6) n=25	2.2 (1.1,4.5) n=11	0.052
BA.1	28	ID50 GMT[1] (95% CI); N	499 (282,885) n=25	14 (9,22) n=11	<0.0001
		Percentage > LoD[2] (95% CI) n/N	96.0 (79.6-99.9); 24/25	27.3 (6.0-61.0); 3/11	<0.0001
		% > PRRT[3] (95% CI) n/N	84.0 (63.9-95.5); 21/25	0.0 (0.0-28.5); 0/11	<0.0001
		D6414G/BA.1[6] (95% CI); N	3.0 (1.2,7.2) n=25	5.6 (1.9,16.4) n=11	0.33
	42	ID50 GMT[1] (95% CI); N	535 (290,988) n=25	16 (9,29) n=8	<0.0001
		Percentage > LoD[2] (95% CI) n/N	100.0 (86.3-100.0); 25/25	37.5 (8.5-75.5); 3/8	0.00024
		% > PRRT[3] (95% CI) n/N	84.0 (63.9-95.5); 21/25	0.0 (0.0-36.9); 0/8	<0.0001
		Fold increase[4] (95% CI) N	1.1 (0.6,1.8) n=25	1.1 (0.8,1.6) n=8	0.85
		D6414G/BA.1[6] (95% CI); N	3.6 (1.8,7.4) n=25	20.0 (6.7,59.1) n=8	0.0092
	180	ID50 GMT[1] (95% CI); N	21 (14,32) n=25	18 (10,33) n=11	0.63
		Percentage > LoD[2] (95% CI) n/N	44.0 (24.4-65.1); 11/25	36.4 (10.9-69.2); 4/11	0.73
		% > PRRT[3] (95% CI) n/N	4.0 (0.1-20.4); 1/25	0.0 (0.0-28.5); 0/11	>0.99
		% decrease from Day 42[5] (95% CI); N	96.0 (92.4,97.9) n=25	4.8 (-91.0,52.5) n=8	<0.0001
		D6414G/BA.1[6] (95% CI); N	27.4 (14.5,51.8) n=25	4.3 (1.3,14.0) n=11	0.0076
BA.4	28	ID50 GMT[1] (95% CI); N	436 (292,649) n=24	13 (9,19) n=8	<0.0001
		Percentage > LoD[2] (95% CI) n/N	100.0 (85.8-100.0); 24/24	25.0 (3.2-65.1); 2/8	<0.0001
		% > PRRT[3] (95% CI) n/N	87.5 (67.6-97.3); 21/24	0.0 (0.0-36.9); 0/8	<0.0001

		D6414G/BA.4[6] (95% CI); N	3.3 (1.5,7.3) n=24	4.7 (1.1,19.1) n=8	0.64
	42	ID50 GMT[1] (95% CI); N	429 (273,675) n=24	14 (5,38) n=4	<0.0001
		Percentage > LoD[2] (95% CI) n/N	100.0 (85.8-100.0); 24/24	25.0 (0.6-80.6); 1/4	0.0012
		% > PRRT[3] (95% CI) n/N	87.5 (67.6-97.3); 21/24	0.0 (0.0-60.2); 0/4	0.0017
		Fold increase[4] (95% CI) N	1.0 (0.5,1.8) n=24	1.4 (0.5,3.8) n=4	0.45
		D6414G/BA.4[6] (95% CI); N	4.4 (2.5,7.6) n=24	10.3 (1.3,80.2) n=4	0.28
	180	ID50 GMT[1] (95% CI); N	37 (24,58) n=22	16 (9,27) n=9	0.012
		Percentage > LoD[2] (95% CI) n/N	72.7 (49.8-89.3); 16/22	33.3 (7.5-70.1); 3/9	0.056
		% > PRRT[3] (95% CI) n/N	0.0 (0.0-15.4); 0/22	0.0 (0.0-33.6); 0/9	>0.99
		% decrease from Day 42[5] (95% CI); N	90.4 (80.9,95.2) n=21	27.4 (-101.2,73.8) n=4	0.0011
		D6414G/BA.4[6] (95% CI); N	14.0 (7.5,26.2) n=22	3.6 (1.1,11.6) n=9	0.037
A.VOI.V2	28	ID50 GMT[1] (95% CI); N	1089 (661,1795) n=25	59 (16,220) n=11	0.00049
		Percentage > LoD[2] (95% CI) n/N	100.0 (86.3-100.0); 25/25	63.6 (30.8-89.1); 7/11	0.0056
		% > PRRT[3] (95% CI) n/N	96.0 (79.6-99.9); 24/25	36.4 (10.9-69.2); 4/11	0.00028
		D6414G/A.VOI.V2[6] (95% CI); N	1.4 (0.6,3.1) n=25	1.4 (0.4,5.1) n=11	>0.99
	42	ID50 GMT[1] (95% CI); N	1057 (468,2387) n=25	80 (25,260) n=11	0.00078
		Percentage > LoD[2] (95% CI) n/N	96.0 (79.6-99.9); 24/25	81.8 (48.2-97.7); 9/11	0.22
		% > PRRT[3] (95% CI) n/N	88.0 (68.8-97.5); 22/25	27.3 (6.0-61.0); 3/11	0.00066
		Fold increase[4] (95% CI) N	1.0 (0.4,2.3) n=25	1.4 (0.4,4.6) n=11	0.64
		D6414G/A.VOI.V2[6] (95% CI); N	1.8 (0.8,4.0) n=25	5.6 (2.0,16.0) n=11	0.073

	180	ID50 GMT[1] (95% CI); N	193 (112,332) n=25	34 (8,155) n=11	0.033
		Percentage > LoD[2] (95% CI) n/N	96.0 (79.6-99.9); 24/25	27.3 (6.0-61.0); 3/11	<0.0001
		% > PRRT[3] (95% CI) n/N	48.0 (27.8-68.7); 12/25	18.2 (2.3-51.8); 2/11	0.14
		% decrease from Day 42[5] (95% CI); N	81.7 (56.0,92.4) n=25	57.2 (-114.0,91.5) n=11	0.32
		D6414G/A.VOI.V2[6] (95% CI); N	3.1 (1.8,5.1) n=25	2.3 (1.5,3.5) n=11	0.37
Gamma	28	ID50 GMT[1] (95% CI); N	1650 (946,2876) n=25	15 (9,27) n=11	<0.0001
		Percentage > LoD[2] (95% CI) n/N	100.0 (86.3-100.0); 25/25	27.3 (6.0-61.0); 3/11	<0.0001
		% > PRRT[3] (95% CI) n/N	96.0 (79.6-99.9); 24/25	0.0 (0.0-28.5); 0/11	<0.0001
		D6414G/Gamma[6] (95% CI); N	0.9 (0.4,1.8) n=25	5.3 (1.8,15.4) n=11	0.0074
	42	ID50 GMT[1] (95% CI); N	1886 (1028,3460) n=25	64 (32,128) n=11	<0.0001
		Percentage > LoD[2] (95% CI) n/N	100.0 (86.3-100.0); 25/25	90.9 (58.7-99.8); 10/11	0.31
		% > PRRT[3] (95% CI) n/N	100.0 (86.3-100.0); 25/25	9.1 (0.2-41.3); 1/11	<0.0001
		Fold increase[4] (95% CI) N	1.1 (0.7,1.9) n=25	4.1 (1.6,10.6) n=11	0.017
		D6414G/Gamma[6] (95% CI); N	1.0 (0.5,2.0) n=25	7.0 (3.7,13.5) n=11	0.00010
	180	ID50 GMT[1] (95% CI); N	451 (178,1141) n=25	33 (7,161) n=11	0.0059
		Percentage > LoD[2] (95% CI) n/N	96.0 (79.6-99.9); 24/25	27.3 (6.0-61.0); 3/11	<0.0001
		% > PRRT[3] (95% CI) n/N	56.0 (34.9-75.6); 14/25	18.2 (2.3-51.8); 2/11	0.067
		% decrease from Day 42[5] (95% CI); N	76.1 (38.9,90.6) n=25	48.0 (-129.9,88.3) n=11	0.35
		D6414G/Gamma[6] (95% CI); N	1.3 (0.6,2.8) n=25	2.4 (1.6,3.4) n=11	0.16
SARS1	28	ID50 GMT[1] (95% CI); N	387 (189,793) n=25	37 (15,92) n=11	0.00019

		Percentage > LoD[2] (95% CI) n/N	88.0 (68.8-97.5); 22/25	54.5 (23.4-83.3); 6/11	0.04
		% > PRRT[3] (95% CI) n/N	80.0 (59.3-93.2); 20/25	27.3 (6.0-61.0); 3/11	0.0064
		D6414G/SARS1[6] (95% CI); N	3.9 (1.4,10.4) n=25	2.2 (0.7,7.2) n=11	0.44
	42	ID50 GMT[1] (95% CI); N	312 (215,453) n=25	39 (18,83) n=11	<0.0001
		Percentage > LoD[2] (95% CI) n/N	100.0 (86.3-100.0); 25/25	72.7 (39.0-94.0); 8/11	0.023
		% > PRRT[3] (95% CI) n/N	80.0 (59.3-93.2); 20/25	9.1 (0.2-41.3); 1/11	0.00011
		Fold increase[4] (95% CI) N	0.8 (0.4,1.7) n=25	1.1 (0.4,2.7) n=11	0.64
		D6414G/SARS1[6] (95% CI); N	6.2 (3.7,10.3) n=25	11.6 (5.1,26.4) n=11	0.18
	180	ID50 GMT[1] (95% CI); N	75 (47,119) n=25	50 (24,106) n=11	0.33
		Percentage > LoD[2] (95% CI) n/N	84.0 (63.9-95.5); 21/25	81.8 (48.2-97.7); 9/11	>0.99
		% > PRRT[3] (95% CI) n/N	36.0 (18.0-57.5); 9/25	9.1 (0.2-41.3); 1/11	0.13
		% decrease from Day 42[5] (95% CI); N	76.0 (58.8,86.0) n=25	-29.3 (-166.5,37.3) n=11	0.00051
		D6414G/SARS1[6] (95% CI); N	7.9 (3.9,16.0) n=25	1.6 (0.5,5.0) n=11	0.018

[1]Geometric Mean Titres (GMTS) of Pseudovirus neutralization assay ID50 with 95% confidence intervals shown within parenthesis followed by the sample size.

[2]Percentage of participants with ID50 above the level of detection (LoD); 95% confidence intervals shown in parenthesis followed by fraction of participants above LoD.

[3]Percentage of participants with ID50 above the putative risk reduction threshold (PRRT) correlating to 80% vaccine efficacy against wild type or Alpha variant (and presuming same threshold holds for other variant specific assays); 95% confidence intervals shown in parenthesis followed by fraction of participants above PRRT.

[4]Geometric means of fold-increases are also reported post-booster (day 42). 95% confidence intervals are shown within parenthesis followed by available sample size.

[5]The percent reduction in the geometric means between two-weeks post-booster (day 42) and six months post-vaccination (day 182). 95% confidence intervals are shown within parenthesis followed by sample size.

[6]The ratio of the geometric means of D6414G and each variant are reported at all time points. 95% confidence intervals are shown within parenthesis followed by sample size.

Supplementary Figure 1: Antibody dependent cellular cytotoxicity (ADCC) relative light unit (RLU) FcγRIIIa signalling AZD1222 vaccinated individuals who were anti-Nucleocapsid IgG seropositive (anti-N IgG+) or seronegative (anti-N IgG-) at time of the first dose of vaccine.

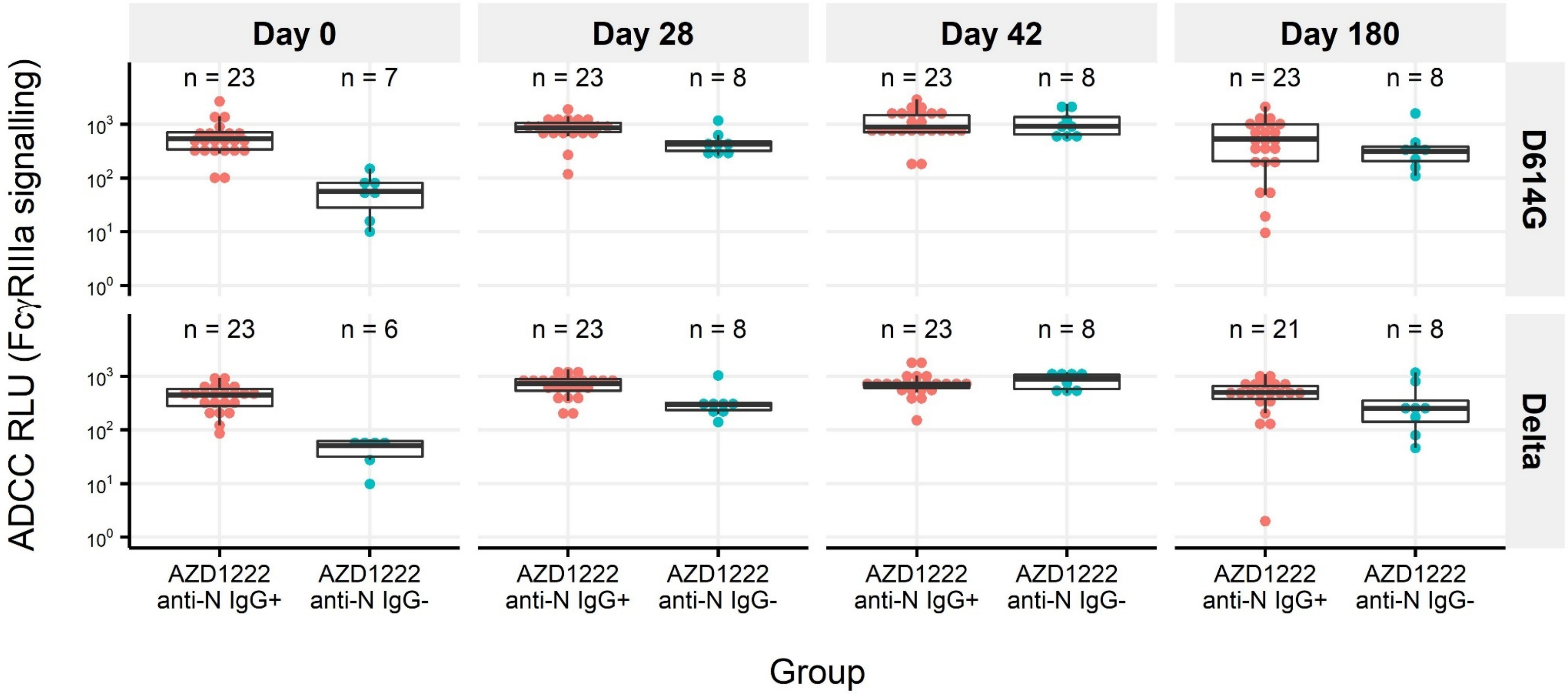

References

1. Madhi SA, Koen AL, Izu A, et al. Safety and immunogenicity of the ChAdOx1 nCoV-19 (AZD1222) vaccine against SARS-CoV-2 in people living with and without HIV in South Africa: an interim analysis of a randomised, double-blind, placebo-controlled, phase 1B/2A trial. *Lancet HIV* 2021; **8**(9): e568-e80.
2. Stadlbauer D, Amanat F, Chromikova V, et al. SARS-CoV-2 Seroconversion in Humans: A Detailed Protocol for a Serological Assay, Antigen Production, and Test Setup. *Curr Protoc Microbiol* 2020; **57**(1): e100.
3. Simani OE, Izu A, Violari A, et al. Effect of HIV-1 exposure and antiretroviral treatment strategies in HIV-infected children on immunogenicity of vaccines during infancy. *Aids* 2014; **28**(4): 531-41.
